# Mutation analysis of pathogenic non-synonymous single nucleotide polymorphisms (nsSNPs) in WFS1 gene through computational approaches

**DOI:** 10.1038/s41598-023-33764-1

**Published:** 2023-04-25

**Authors:** Jing Zhao, Siqi Zhang, Yuan Jiang, Yan Liu, Qingwen Zhu

**Affiliations:** 1 Department of Otolaryngology, The Third Hospital of Hebei Medical University, Hebei, China; 2 Department of Otolaryngology, The Second Hospital of Hebei Medical University, Hebei, China

**Keywords:** Genetics research, Disability, Mutation

## Abstract

A single base changes causing a change to the amino acid sequence of the encoded protein, which is defined as non-synonymous single nucleotide polymorphisms (nsSNPs). Many of the nsSNPs can cause disease, and these nsSNPs are considered as pathogenic mutations. In the study, the high-risk nsSNPs of WFS1 and their influence on the structure and function of wolframin protein were predicted by multiple bioinformatics software. We obtained 13 high-risk nsSNPs of WFS1. All the 13 high-risk nsSNPs are highly conserved residues with a conservative score of 9 or 8 and mostly may cause a decrease in protein stability. The high-risk nsSNPs have an important effect on not only amino acid size, charge and hydrophobicity, but also protein's spatial structure. Among these, 11 nsSNPs had been previously published or cited and 2 nsSNPs (G695S and E776K) had not been reported to date. The two novel variants increased or decreased hydrogen bonds. In conclusion, through different computational tools, it is presumed that the mechanism of pathogenic WFS1 nsSNPs should include the changes of physicochemical properties, significant structural changes and abnormal binding with functional partners. We accomplished the computational-based screening and analysis for deleterious nsSNPs in WFS1, which had important reference value and could contribute to further studies of the mechanism of WFS1 related disease. The computational analysis has many advantages, but the results should be identified by further experimental studies in vivo and in vitro.

## Introduction

Single nucleotide polymorphisms (SNPs) are widely known to be the most common genetics variant of human genome, defined as a substitution of a single nucleotide occurring at a specific position in the genome. The frequency of each of SNPs in the general population is more than 1%. The SNPs generally have an important effect on many genetic diseases^1^. SNPs include two categories: synonymous or non-synonymous SNPs. Due to amino acid substitutions, the non-synonymous coding SNPs (nsSNPs) may significantly influence the protein function and structure, thus the disease phenotype. We should deeply delve into how the nsSNPs affect the function of proteins to have a better knowledge of the genetic basis of human intricate diseases such as hearing loss.

The Wolfram syndrome type 1 (WFS1) gene maps to chromosome 4p16.1, and its 3628 bp coding sequence is arranged in eight exons, the first of which is non-coding (Fig S1a). The positions of the exon regions were predicted based on the study of Eleonora Panfili^2^. Mutations are mostly found to occur in exon 8, which is the largest exon, containing 2.6 kb of DNA. The wolframin protein is a 100.29-kDa protein containing 890 amino acids and encoded by WFS1 gene, predominantly localizing in endoplasmic reticulum (ER). The hypothetical structure of the wolframin protein is shown in Fig S1b. The positions of the transmembrane regions were annotated according to the study of Dewi Astuti^3^. The protein consists of three parts: a hydrophilic N-terminus of about 300 residues, a hydrophilic C-terminus of 240 residues, and a central hydrophobic domain of 350 residues, containing nine transmembrane regions.

The WFS1 pathogenic variants mainly cause Wolfram syndrome^4^ and NSHL^5^, whose common feature is hearing loss. So far, more than 490 variants of WFS1 gene have been reported. It is found that the most common type of WFS1 variants is missense mutation, accounting for about 80%. The pathogenic missense mutations are mainly located at exon 8. Numerous of nsSNPs in WFS1 have been found. Although they may have destructive effect on the function of wolframin protein, it is not only time-consuming but also expensive to deeply explore their functional effect. It is worthwhile to use different bioinformatics tools to analyze the high-risk nsSNPs. Our study focused on the relationship among these nsSNPs and protein function in depth.

## Materials and methods

### Data mining

Three databases retrieved WFS1 SNPs for subsequent computational analysis, including the ClinVar database (https://www.ncbi.nlm.nih.gov/clinvar), Deafness variation database (https://deafnessvariationdatabase.org/) and dbSNP database (http://www.ncbi.nlm.nih.gov/projects/SNP/). We used the ANNOVAR to identify the WFS1 SNPs. According to its instruction, we used the 1000 Genomes Project (2014 Oct) annotations through changing command line argument to 1000g2014oct.

### Prediction of high-risk nsSNPs in WFS1

To assess the potential effect of SNPs in the WFS1, we performed analyses utilizing a range of database servers. When all the computational tools predict one nsSNP is deleterious, we consider it as the high-risk nsSNP, which is highly likely to have harmful effects on the function of protein and even lead to diseases.

Sorting intolerant from tolerant (SIFT) (http://sift.jcvi.org/) and Protein variation effect analyzer (PROVEAN) (http://provean.jcvi.org) can predict the potential influence of an amino acid substitution in a protein according to the sequence homology^6^. Polymorphism phenotyping V2 (PolyPhen-2) (http://genetics.bwh.harvard.edu/pph2/) can calculate the potential functional effect of amino acid substitutions from its individual characteristics via Naïve Bayes classifier^7^. Likelihood Ratio Test (LRT) uses the statistical method of likelihood ratio test to make predictions by analyzing the conservation of amino acids^8^. Unlike SIFT and PloyPhen-2, LRT does not need to analyze the evolutionary distance between homologous protein sequences to predict amino acid conservation, and has a wider range of applications. Functional Analysis through Hidden Markov Models (FATHMM) (http://fathmm.biocompute.org.uk/inherited.html) can predict the impact of missense mutations on the function of protein with optional species-specific weights^9^. Its MKL algorithm can be used to predict both coding and non-coding variants. Mutation Taster (http://www.mutationtaster.org/ChrPos.html) is an analysis tool which have recruits several biomedical databases, and predict that the mutation is a polymorphism or disease causing through a naive Bayes classifier2^10^. Based on evolutionary conservation of the mutant amino acid in protein homologs, Mutation Assessor (http://mutationassessor.org/r3/) can assess the functional influence of nsSNPs^11^. Protein variation effect analyzer Variant Effect Scoring Tool 3 (VEST3) (http://karchinlab.org/apps/appVest.html) can predict the functional influence of variants according to the probability of missense mutations causing disease^12^. There are also following six comprehensive prediction tools using machine learning and other related algorithms to score the pathogenicity of a SNP and other variants: CADD^13^, DANN^14^, Meta SVM, MetaLR^15^, M-CAP^16^ and REVEL^17^.

### Prediction of stability of mutant proteins

MUpro (http://mupro.proteomics.ics.uci.edu) can predict protein stability changes without tertiary structures with two machine learning methods: Neural Networks and Support Vector Machines^18^. The confidence score is between 1 and -1. The bigger the absolute value, the more confident the prediction is. I-Mutant2.0 (https://folding.biofold.org/i-mutant/i-mutant2.0.html) can evaluate the change of protein stability upon single site mutation starting from the protein sequence or structure. INPS-MD (http://inpsmd.biocomp.unibo.it), also named as Impact of Non-Synonymous Mutations on Protein Stability-Multi Dimension, is a web server devised to prediction of protein stability change upon single point mutation^19^. The iStable (http://predictor.nchu.edu.tw/istable/indexSeq.php) is a comprehensive predictor of protein stability change after single mutation^20^.

### Evolutionary conservation analysis of nsSNPs

The ConSurf server (http://consurf.tau.ac.il) can calculate the conservation score to estimate the conservation of amino acids in evolution through a maximum likelihood (ML) method or an empirical Bayesian method^21^. The score between 7 and 9 is considered evolutionarily conservative.

### Prediction of secondary structure and membrane protein topology

SOPMA is also named as Self-Optimized Prediction Method with Alignment based on the homologue method. It can predict the secondary structure of protein through five independent algorithms^22^. TMHMM Server 2.0 (https://services.healthtech.dtu.dk/service.php?TMHMM-2.0) is an online tool, based on a hidden Markov model (HMM) approach, for prediction of transmembrane structures in proteins.

### Model building and evaluation of wolframin protein, analysis of mutation-induced structural changes

The Robetta (http://robetta.bakerlab.org/) is a prediction server of protein structure, using the Robetta fragment-insertion method. It can predict a full chain protein structure automatically for ab initio and comparative modeling. The three-dimension (3D) model of the wolframin protein was built by Alphafold2, with the template performed using Robetta server. PyRAMA was used to geometrically evaluated the modeled 3D structure by calculating the Ramachandran plot. Structural presentation of wild and mutant proteins was made by using PyMOL programs. Cartoon drawings of the structures were obtained. PyMOL software was used to label native as well mutant amino acids and present the hydrogen bond between them. Modeled mutant proteins was superimposed with PyMOL on the wild protein for comparison of three-dimensional structure of wild and mutant proteins. As an online web service, HOPE (http://www.cmbi.umcn.nl/hope) can analyze the impact of a given mutation on the protein structure. The Accessible Surface Area and Accessibility Calculation for Protein (ver. 1.2) online server can calculate the solvent-accessible surface areas of 890 amino acids of wolframin protein(http://cib.cf.ocha.ac.jp/bitool/ASA/)^23^.

### Analysis of interaction network

STRING (https://cn.string-db.org/), also named as Search Tool for the Retrieval of Interacting Genes/proteins, is a web based server exploring the target gene interaction network with other proteins. The high confidence level is above 0.700.

## Results

### Dataset

**Figure 1:**
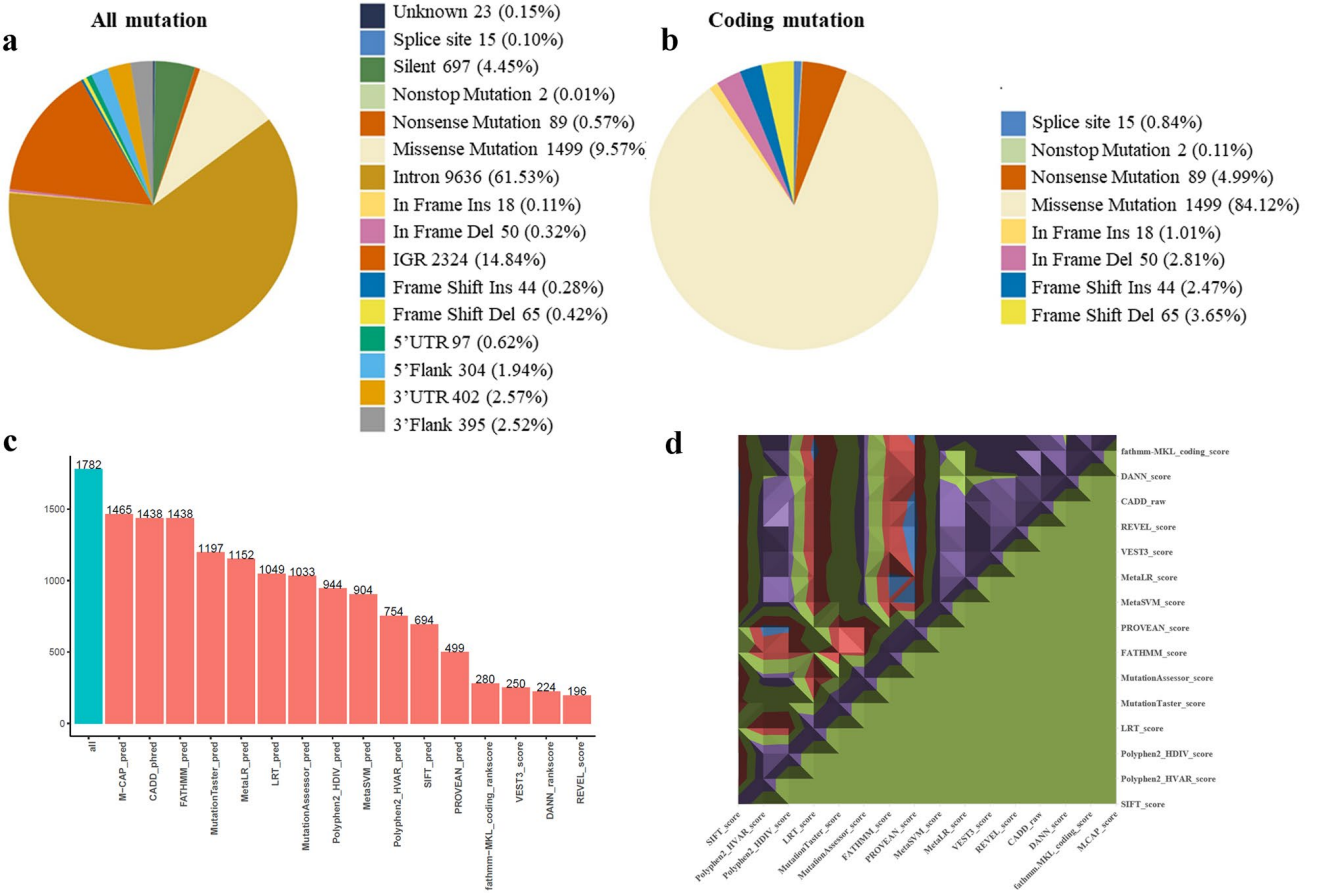
Distribution of mutations and prediction of damaging nsSNPs in WFS1 gene. (**a**) Distribution of all types of mutations in WFS1 gene including splice site, silent, intron, nonsense, missense, 5′UTR and 3′UTR domains, frame shift and so on. (**b**) Distribution of all types of coding mutations in WFS1 gene. (**c**) Number of high-risk nsSNPs in WFS1 predicted by computational tools. (**d**) A surface chart representing the correlations among the harmful predictions by multiple bioinformatics tools in WFS1 gene.

Firstly, 13,521 SNPs were retrieved from the dbSNP database, 1146 from the ClinVar database, and 7203 from the Deafness variation database. Secondly, we eliminated the duplicate SNPs and obtained 15,660 WFS1 SNPs. The introns occupy 61.53% of 15,660 SNPs (Fig. 1a). At last, after removing the noncoding SNPs and manual screening, there were 1782 WFS1 SNPs (Fig. 1b). In the SNPs which occur at coding region of WFS1 gene, the proportion of missense SNPs is 84.12%.

### Identification of the pathogenicity of nsSNPs

We used fourteen computational tools to predict whether every one of the nsSNPs is deleterious or not (Table S1). The number of deleterious nsSNPs predicting by each software was shown in Fig. 1c. The screening conditions predicted as harmful by each software are as follows: "Deleterious" by M-CAP, FATHMM, MetaLR, LRT, MetaSVM and PROVEAN; “Probably damaging” or “Possibly damaging” by Polyphen2; "Damaging” by SIFT; "High” or “Medium” by MutationAssessor; "Disease causing automatic” or “Disease causing” by Mutation Taster2; rank score higher than 0.9 by FATHMM -MKL and DANN; score higher than 25 by CADD_Phred; score higher than 0.9 by VEST3 and REVEL. There were 13 nsSNPs which considered as the highly harmful nsSNPs by all the computational tools. As shown in Fig. 1d, the darkest purple region was highlighted and had the positive correlation with highly harmful nsSNPs in WFS1. The detailed results are highlighted in Table 1. Finally, the 13 nsSNPs were considered high risk and selected for in-depth analysis.

**Table 1:** Cumulative prediction of damaging nsSNPs in WFS1.

Substituent	Nucleotide Variation	SNP ID	SIFT Score	Polyphen2 HDIV score	Polyphen2 HVAR score	LRT score	MutationTaster score	Mutation Assessor score	FATHMM score	FATHMM-MKL rankscore	PROVEAN score	VEST3 score	MetaSVM score	MetaLR score	M-CAP score	REVEL score	CADD phred	DANN rankscore
G107R	G319C		0.003	1	0.996	0	1	2.585	− 3.78	0.956	− 3.97	0.924	1.042	0.919	0.854	0.95	26	0.99
G494R	G1480C	rs760692398	0.002	1	0.994	0	1	2.85	− 2.73	0.986	− 5.82	0.972	0.869	0.836	0.814	0.943	25.9	0.966
A684T	G2050A	rs1412819148	0	1	0.997	0	1	2.84	− 4.18	0.901	− 3.03	0.937	1.087	0.943	0.835	0.964	28	0.988
G695S	G2083A	rs1252460131	0	1	0.995	0	1	2.89	− 4.19	0.901	− 5.51	0.955	1.086	0.942	0.806	0.98	27.9	0.93
G702S	G2104A	rs71532862	0	1	1	0	1	2.91	− 4.29	0.901	− 5.99	0.989	1.1	0.954	0.892	0.978	29.2	0.924
L723P	T2168C		0.001	1	0.994	0	1	2.825	− 4.43	0.93	− 6.06	0.969	1.095	0.946	0.902	0.943	25.5	0.923
P724L	C2171T	rs28937890	0.002	1	1	0	1	2.89	− 4.34	0.936	− 9.92	0.988	1.1	0.954	0.924	0.948	31	0.967
R732H	G2195A	rs149013740	0.018	1	0.995	0	1	2.6	− 4.4	0.967	− 3.37	0.916	1.094	0.95	0.816	0.971	32	0.997
G736S	G2206A	rs71532864	0	1	1	0	1	2.91	− 4.24	0.967	− 5.65	0.99	1.095	0.946	0.893	0.968	32	0.929
G736R	G2206C	rs71532864	0	1	1	0	1	2.91	− 4.25	0.977	− 7.6	0.988	1.099	0.952	0.922	0.987	29.4	0.963
E776K	G2326A	rs1421068689	0.005	0.998	0.956	0	1	2.51	− 3.67	0.991	− 3.38	0.927	1.029	0.915	0.805	0.963	29.1	0.984
L829P	c.T2486C	rs104893883	0.001	1	1	0	1	2.42	− 3.66	0.985	− 4.79	1	0.952	0.846	0.876	0.916	25.7	0.947
P885L	c.C2654T	rs372855769	0	1	0.999	0	1	2.51	− 4.4	0.926	− 7.69	0.946	1.052	0.909	0.92	0.932	28.6	0.969

### Changes of protein stability after mutations

**Figure 2:**
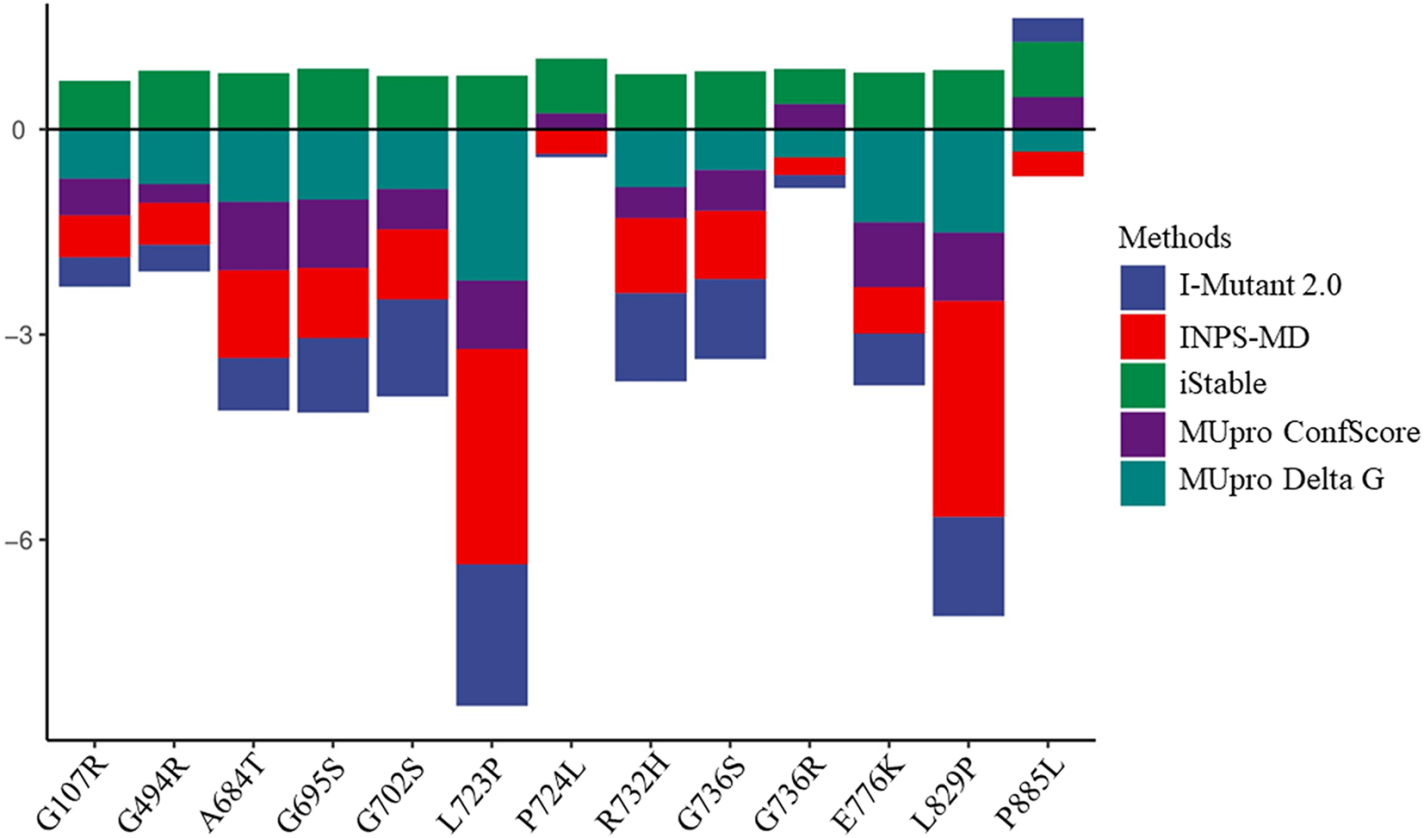
MUpro, I-Mutant 2.0, INPS-MD and iStable predicted the change of protein stability resulted from nsSNPs.

The effect of 13 nsSNPs on protein stability were predicted by MUpro, I-Mutant 2.0, INPS-MD and iStable software (Fig. 2 and Table 2). INPS-MD software predicted that all 13 nsSNPs resulted in decreased protein stability. MUpro software, I-Mutant 2.0 and iStable software predicted 10, 10 and 11 nsSNPs leading to a decline in stability of wolframin protein, respectively. Meanwhile, the stability of protein had a sharp decline after the L723P and L829P mutations, because their total score was both below -6. Moreover, P724L and P885L were predicted to increase the stability of wolframin protein by three software tools.

**Table 2:** Validation result of protein stability change by using MUpro, I-Mutant 2.0(Seq), INPS-MD and iStable.

Substitutions	MUpro				I-Mutant 2.0(Seq)		INPS-MD		iStable	
	Confidence score	Prediction	Delta G	Prediction	DDG	Prediction	DDG	Prediction	Confidence score	Prediction
G107R	− 0.53681855	Decrease	− 0.719856	Decrease	− 0.43	Increase	− 0.614571	Decrease	0.711645	Decrease
G494R	− 0.27432649	Decrease	− 0.799365	Decrease	− 0.39	Decrease	− 0.614996	Decrease	0.859133	Decrease
A684T	− 1	Decrease	− 1.059934	Decrease	− 0.77	Decrease	− 1.28314	Decrease	0.823745	Decrease
G695S	− 1	Decrease	− 1.025896	Decrease	− 1.09	Decrease	− 1.02497	Decrease	0.888069	Decrease
G702S	− 0.58902382	Decrease	− 0.87166	Decrease	− 1.42	Decrease	− 1.02497	Decrease	0.781893	Decrease
L723P	− 1	Decrease	− 2.211354	Decrease	− 2.07	Decrease	− 3.14935	Decrease	0.788351	Decrease
P724L	0.21392985	Increase	0.021993	Increase	− 0.05	Increase	− 0.357545	Decrease	0.799145	Increase
R732H	− 0.45171425	Decrease	− 0.845002	Decrease	− 1.29	Decrease	− 1.09836	Decrease	0.80706	Decrease
G736S	− 0.59692055	Decrease	− 0.594136	Decrease	− 1.17	Decrease	− 0.997424	Decrease	0.850104	Decrease
G736R	0.37352591	Increase	− 0.41075	Decrease	− 0.19	Decrease	− 0.257352	Decrease	0.510684	Decrease
E776K	− 0.94924869	Decrease	− 1.358258	Decrease	− 0.76	Decrease	− 0.677756	Decrease	0.830963	Decrease
L829P	− 1	Decrease	− 1.508759	Decrease	− 1.45	Decrease	− 3.16076	Decrease	0.868579	Decrease
P885L	0.47334686	Increase	− 0.326501	Decrease	0.35	Increase	− 0.357545	Decrease	0.804748	Increase

### Evolutionary Conservation Analysis

**Figure 3:**
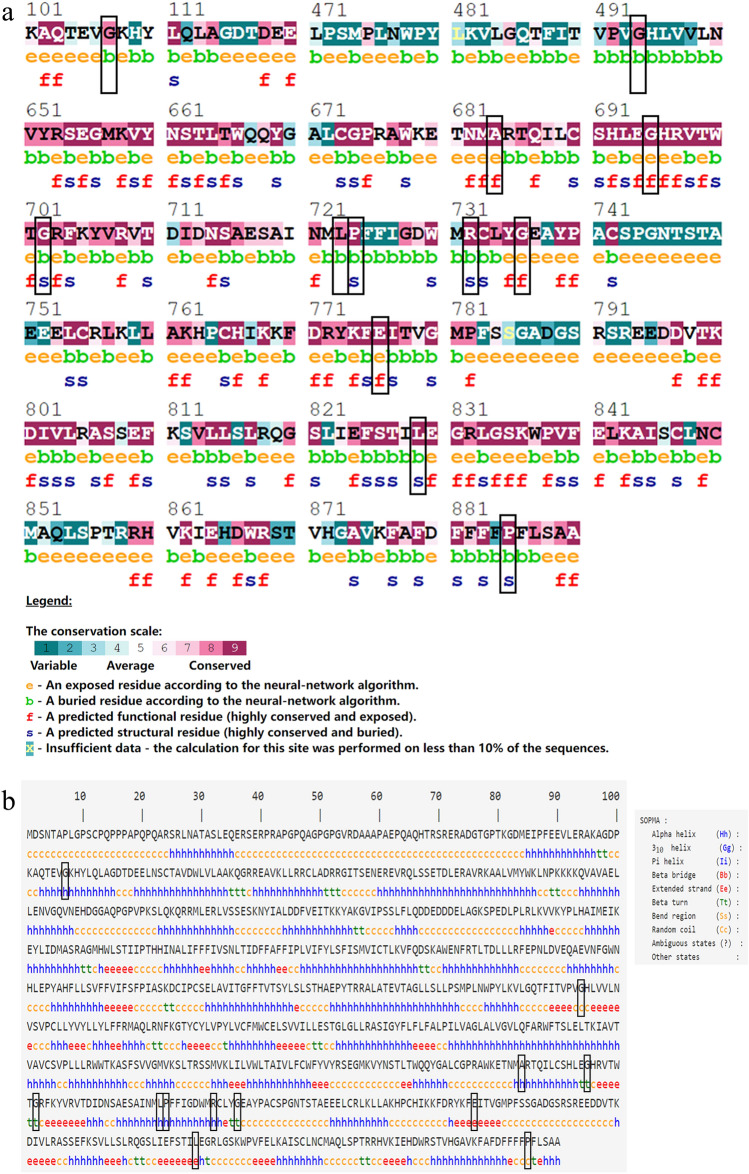
Combined figures. (**a**) The prediction results of ConSurf software about the evolutionary conservation of WFS1. The mutant amino acid sites are highlighted by black boxes. The color-coding bar represents the conservation score. (**b**) The secondary structure of wolframin protein according to SOPMA analysis. The high-risk nsSNPs are highlighted by black boxes.

According to the result of the ConSurf analysis, more than half of the 890 positions of wolframin protein were evolutionarily conserved, scoring between 7 and 9 (Fig. 3a). It was demonstrated that G107R, G494R, L723P had a conservation score of 8 and A684T, G695S, G702S, P724L, R732H, G736S, G736R, E776K, L829P, P885L had a conservation score of 9.

### The secondary structure, and transmembrane helices prediction of the wolframin protein

**Figure 4:**
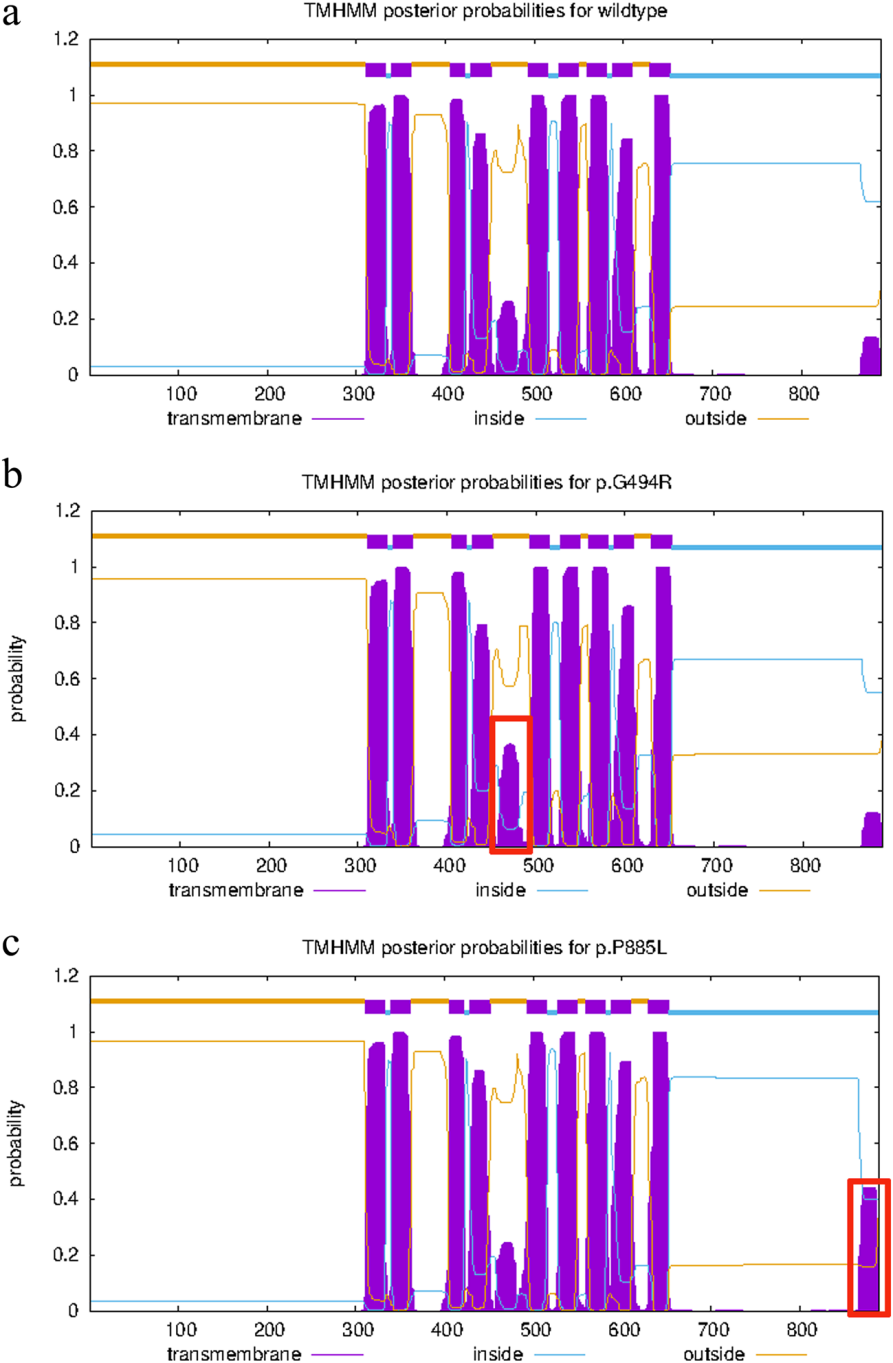
The nine transmembrane regions in WFS1 shown by TMHMM server. The red boxes indicate the change of transmembrane probability due to mutant amino acid (**a** is the wild type, **b** is the G494R mutant type, and **c** is the P885L mutant type).

The secondary structure of the wolframin protein was predicted by SOPMA (Fig. 3b). Four secondary structures composed the wolframin protein with 890 amino acids (Fig S2). The alpha helix consisted of 442 amino acids (accounting for 49.66%), the beta turn consisted of 44 amino acids (4.94%), the beta sheet consisted of 98 amino acids (11.01%), and the random coil consisted of 306 amino acids (34.38%). We used the TMHMM to characterize the amino acid of WFS1 for their inside/outside of membrane region and transmembrane region and investigated the effect of mutations of high-risk nsSNPs. It was showed that there were nine transmembrane regions in WFS1 by using TMHMM server (Fig. 4a–c). The G494R and P885L mutation significantly increased the probability that the corresponding amino acid site is located at transmembrane region. However, none of the nsSNPs resulted in changes in the structure of the wolframin transmembrane region. Notably, most pathogenic variants were found in the C-terminal region of wolframin rather than the transmembrane domain.

### Protein modeling of wolframin and analysis for the structural effects of mutation

**Figure 5:**
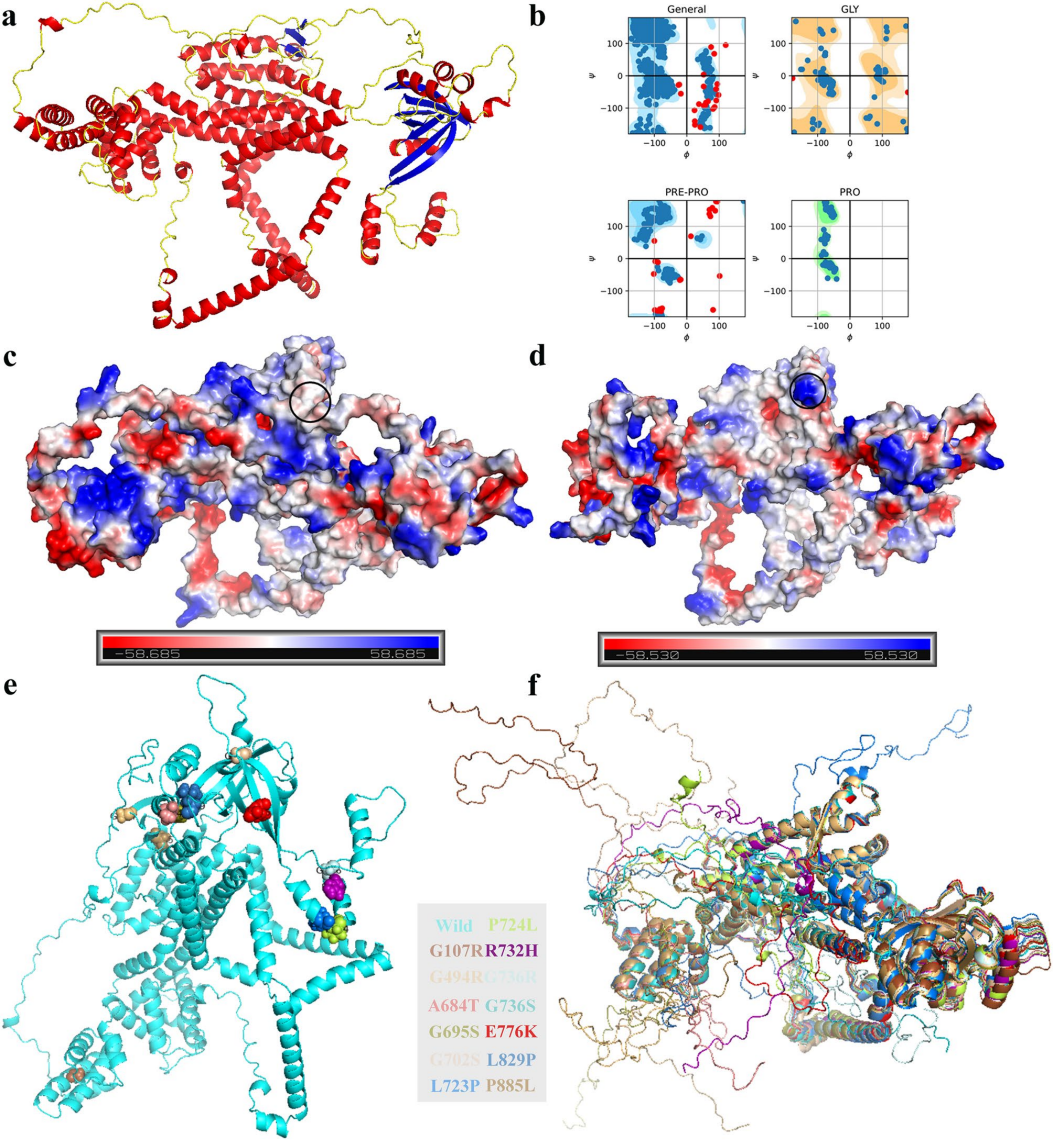
Protein structure predicted by the PyMOL. (**a**) The protein structure of the wild type wolframin (Red: alpha helix; Blue: beta sheet; Yellow: random coils and other structures). (**b**) A validation of 3D models by Ramachandran plot. (**c**) A qualitative electrostatic representation of wolframin protein generated by PyMOL. Protein contact potentials can be represented by displaying virtual (false) red/blue charged smooth surfaces on wolframin protein. (**d**) A qualitative electrostatic representation of mutant G494R protein generated by PyMOL. The black circle indicates the position of amino acid 494. (**e**) The wildtype protein model highlighting substitution regions. (**f**) Mutation-induced structural changes in WFS1. It shows superimposed view of wolframin protein in wild and mutant state.

The 3D structures of wolframin and its mutant proteins were predicted by Robetta server (Fig. 5a). The Ramachandran analysis was carried out for wolframin protein. The residues of the wild type protein were greater than 90% in most favored and allowed region, which showed the structure was usual (Fig. 5b). According to the comparison of the qualitative electrostatic representation of wild and mutant G494R proteins, it was found that the G494R mutation changed the charge of the amino acid at this site from neutral to positive (Fig. 5c,d). Figure 5e exhibited the wildtype protein model highlighting substitution regions. Modeled mutant proteins was superimposed on the wild protein by PyMOL for comparison of three-dimensional structure of wild and mutant proteins (Fig. 5f). Almost all nsSNPs resulted in the structural drifting, further confirming by energy refinement. We calculated root-mean-square deviation (RMSD) values for all mutant models (Table 3). The value means the average distance of α‐carbon backbones between mutant and wild model. The structure deviation between mutant and wild protein was positively correlated with RMSD value. The model of the G107R mutation had the greatest deviation with 1.730B RMSD value followed by P885L, G702S, L829P, G494R and P724L with 1.573B, 1.504B, 1.496B, 1.469B and 1.468B RMSD values, respectively. Others had slight changes including G736S (1.367B RMSD), L723P (1.133B RMSD), and A684T (1.106B RMSD).

**Table 3:** Evolutionary conservativeness analyses and protein prediction of WFS1 high-risk pathogenic nsSNPs.

Amino acid change	Domain	Consurf score	SOPMA predicting secondary structure	RMSD value	Change of size	Change of charge	Change of Hydrophobicity
G107R	N-terminal	8	Alpha helix	1.730	M > W	Neutral → positive	Decrease
G494R	TM5	8	Random coil	1.469	M > W	Neutral → positive	Decrease
A684T	C-terminal	9	Alpha helix	1.106	M > W		Decrease
G695S	C-terminal	9	Beta turn	0.865	M > W		
G702S	C-terminal	9	Beta turn	1.504	M > W		
L723P	C-terminal	8	Alpha helix	1.133	M < W		
P724L	C-terminal	9	Alpha helix	1.468	M > W		
R732H	C-terminal	9	Alpha helix	0.989	M < W	Positive → neutral	
G736S	C-terminal	9	Beta turn	1.367	M > W		
G736R	C-terminal	9	Beta turn	0.977	M > W	Neutral → positive	Decrease
E776K	C-terminal	9	Extended strand	0.769	M > W	Negative → positive	
L829P	C-terminal	9	Extended strand → Alpha helix	1.496	M < W		
P885L	C-terminal	9	Random coil → Alpha helix	1.537	M > W		

*TM* transmembrane, *W* wild type, *M* mutant type.

**Figure 6:**
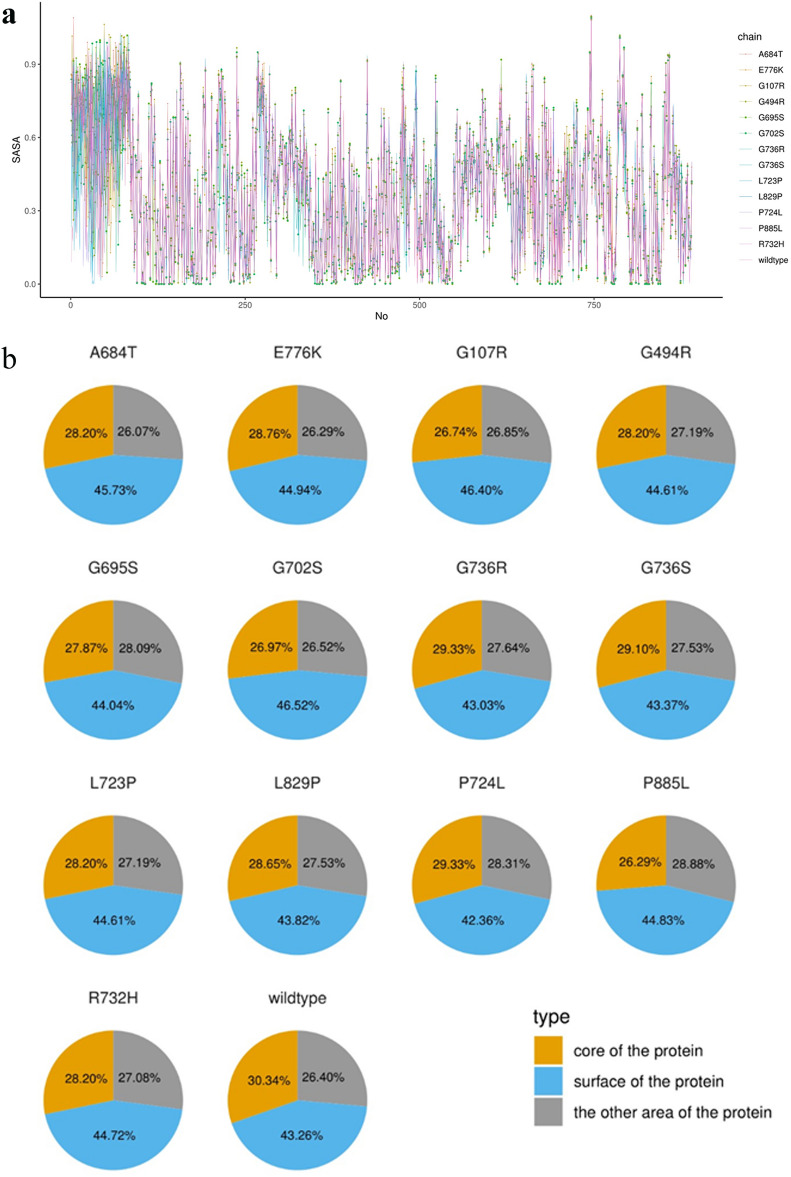
The solvent accessible surface areas of the wolframin protein. (**a**) The bottom panels describe per-residue SASA of wolframin protein and mutant proteins. (**b**) The distribution about the SASA of all 890 amino acids in wild and mutant proteins.

The changes of amino acid substitutions on the size, hydrophobicity, structure and so on of wolframin were predicted by HOPE (Table 3). All 13 nsSNPs brought about changes in size of amino acids (10 larger and 3 smaller) and 5 nsSNPs resulted in change of charge. Besides, 4 nsSNPs decreased the hydrophobicity. It is speculated that these changes can lead to changes of intramolecular interactions so that affect the function of wolframin protein. The changes of solvent accessible surface areas (SASA) were analyzed by the Accessible Surface Area and Accessibility Calculation for Protein (ver. 1.2) online server, which is considered as an important factor in protein folding and stability studies. According to SASA analysis, the similar residual fluctuations were shown between the wild and mutant protein (Fig. 6a). SASA parameter is proven that the protein is accessible to other ligand and/or proteins. As shown in Fig. 6b, there were 385 (43.26%) amino acids on the surface, 270 (30.34%) in the core and 235 (26.40%) in other parts of wolframin protein. The proportion of protein surface amino acids was increased except for P724L.The proportion of protein core amino acids was decreased in all 13 WFS1 high-risk pathogenic nsSNPs mutations, meaning that more amino acids were exposed and eventually may have harmful effects on interaction with other proteins.

**Figure 7:**
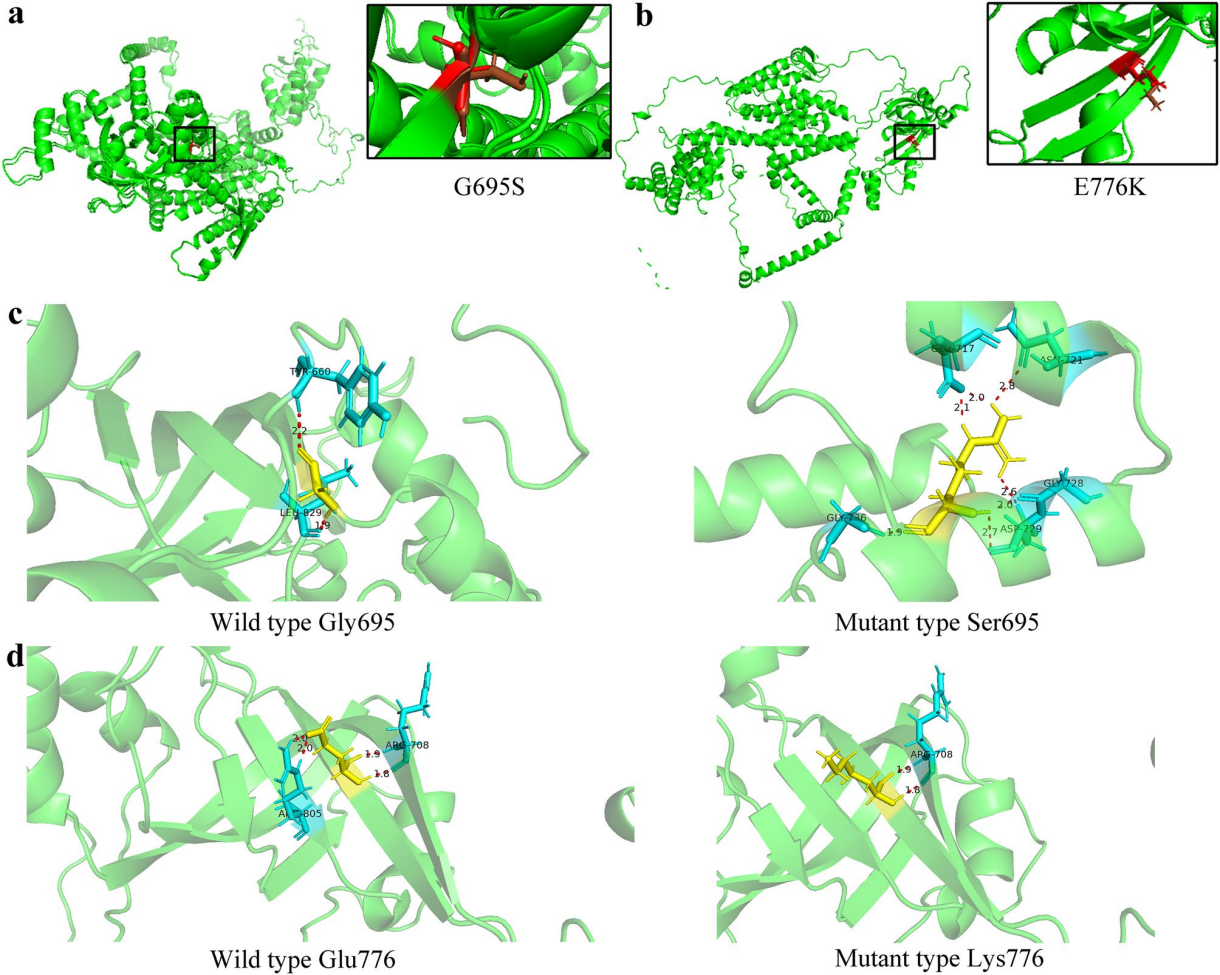
3D protein modeling of WFS1 variants at amino acid residue 695 and 776. (**a,b**) Predicted structures depict the changes of mutant wolframin protein with the amino acid change G695S, and E776K. Red and brown structures indicate differences between wild and mutant type. (**c,d**) The change of hydrogen bond between amino acids before and after mutation. The proteins are shown as cartoon. Amino acids at the mutated site are highlighted in yellow and the interacting amino acids are highlighted in blue (red dotted line: the hydrogen bond).

In the next step, we selected two novel nsSNPs (G695S and E776K) that have not been reported (Fig. 7a,b). All the novel variants increased or decreased hydrogen bonds (Fig. 7c,d). In the wild type, Gly695 has a hydrogen bond with Tyr660 and Leu829, respectively. In the mutant type G695S, the original hydrogen bond distances are changed and a hydrogen bond between Ser695 and Glu694 is added. Wild type has two hydrogen bonds between Glu776 and Arg708, Arg805, respectively. The variant E776K eliminates hydrogen bonds between Lys776 and Arg805. Changes in hydrogen bonds may influence the stability and intramolecular interactions of the wolframin protein, then causing diseases.

### Protein–protein interaction and functional characterization

**Figure 8:**
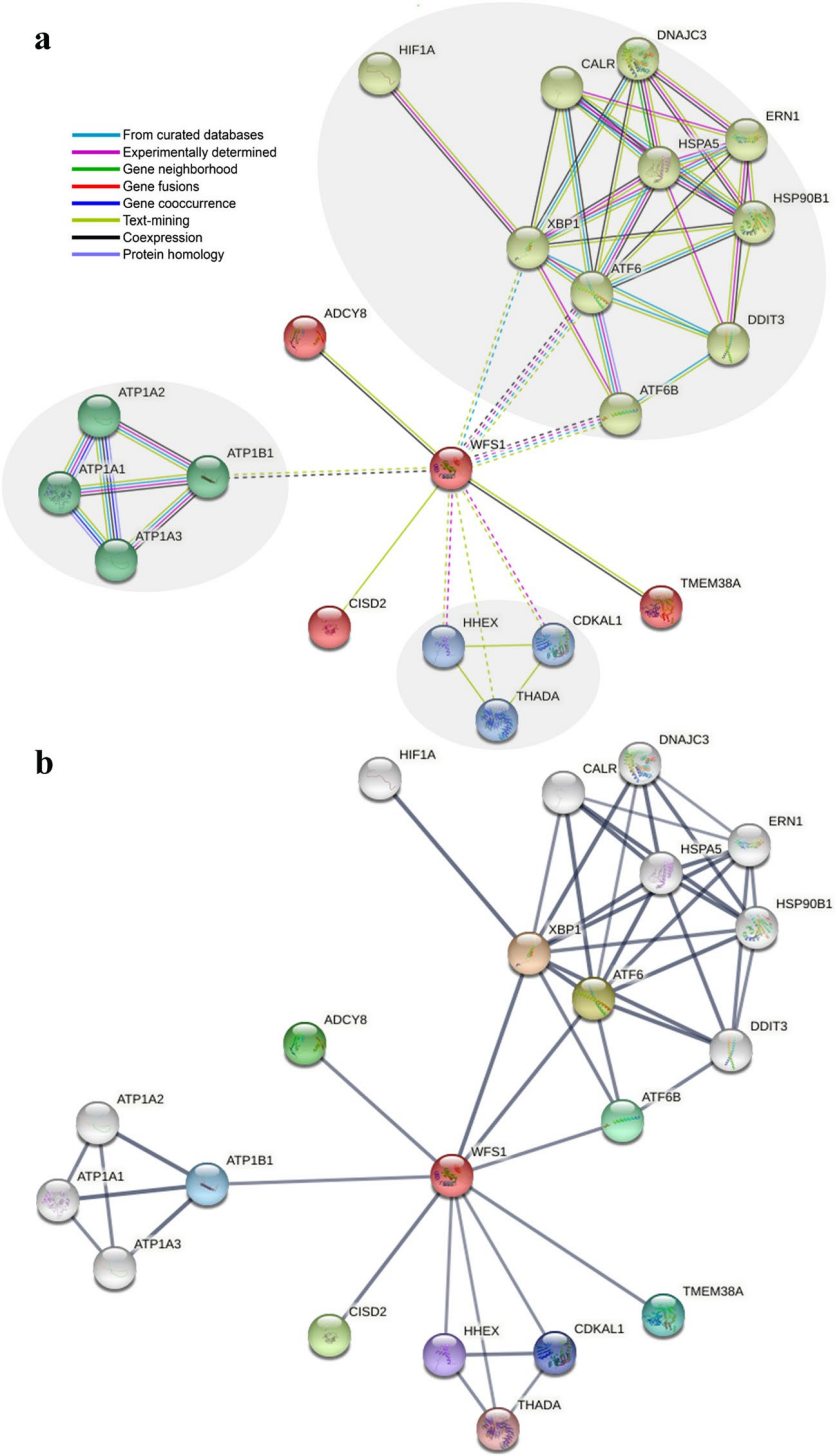
The interaction network analysis of WFS1. (**a**) Line color indicates the type of interaction evidence. The three grey areas indicate the large cluster with functional proteins (edges between clusters: dotted line). (**b**) Line thickness indicates the strength of data support.

We could have a knowledge of the interacting partners of WFS1 through using STRING database (Table S3). At high confidence score 0.700, the number of average node degree, nodes and interaction number of edges were 4.57, 21 and 48 respectively (Fig. 8a,b).

It is shown that WFS1 interacts with ATP1A1, ATP1A2, ATP1A3 and ATP1B1, then connecting with Na/K-ATPase. It is also named as sodium/potassium adenosine triphosphatase or ATP1A protein. ATP1A consists of a α subunit and a β subunit and plays an important part in keeping the electrochemical gradient on the cell membrane. ATP1A1, ATP1A2 and ATP1A3 belong to the α subunit, and ATP1B1 belongs to the β subunit. Furthermore, WFS1 interacted with ATF6, ATF6B and XBP1, which were involved in the unfolded protein response (UPR) and endoplasmic reticulum stress (ERS), and maintained interactions with Ca^2+^-associated folding factors (HSPA5, HSP90B1/GRP94, and CALR) and other chaperones (ERN1 and DNAJC3). Among the other interacting proteins of the WFS1, CISD2 and TMEM38A play a vital part in regulation of cytosolic Ca^2+^ homeostasis, and ADCY8 is essential for activating the glucose-induced signaling pathways in beta cells. WFS1 also interacts with the insulin release-related proteins (HHEX and CDKAL1), which are strongly associated with genetic risk variants for diabetes.

## Discussion

We screened out 13 high-risk nsSNPs of WFS1 gene, in which 11 nsSNPs had been reported in the literature. It is noteworthy that one nsSNP (L829P) is associated with non-syndromic hearing loss and eight nsSNPs (G107R, A684T, G702S, L723P, P724L, G736S, G736R and P885L) are associated with Wolfram syndrome (WS)^24–27^. The G494R and R732H were reported as the variants of uncertain significance. Wolfram syndrome is an autosomal recessive disorder, and its clinical features are diabetes insipidus, diabetes mellitus, optic atrophy and deafness. The mutational studies of Wolfram syndrome reported most pathogenic variants were located in transmembrane region and carboxy tail of wolframin protein, inside exon 8. However, the nsSNP (c.319G > C, p.G107R) was detected in two siblings from Southern Italy with Wolfram syndrome (WS), inside exon 4^28^.

We obtained two novel nsSNPs (G695S and E776K) from WFS1 high-risk nsSNPs. We speculate that they are highly likely to be pathogenic mutations, because:(1) they were all predicted to be highly harmful by all predictive tools; (2) they all had highly score 9 in conservation analysis; (3) they could all lead to decreasing protein stability; (4) they could all cause changes in amino acid properties and tertiary structure. However, clinical literature reports and other evidence are needed to verify their pathogenicity.

In order to further explore the potential pathogenic mechanism of WFS1 high-risk nsSNPs, we analyzed the stability, conservation, the physical and chemical properties, tertiary structure through many software tools. The conservation analysis showed that all 13 high-risk nsSNPs were highly conserved in the WFS1, regarded as massively damaging, because the residues of the conserved domain have an important effect on biological process such as interactions among proteins. The wild-type residues of G107R, G494R, G695S, G702S, G736S and G736R are glycine, which is flexible enough to make torsion angles. Their mutant residues cause torsion angles to be unusual so that force the local backbone into an incorrect conformation and disturb the local structure. As the wild-type residues of P724L and P885L, proline has a very rigid structure, thus inducing a special backbone conformation which might be required at corresponding positions. These mutations with leucine residue maybe disturb the local structure and function of wolframin protein.

According to multiple studies, the wolframin protein have a vital function on the following aspects: (1) interaction with Na+/K + ATPase β subunit^29^; (2) regulation of the ER stress response^30^;(3) regulation of the cellular calcium homeostasis^31^; and (4) regulation of insulin production and secretion from pancreatic β-cells^32^. The same conclusion can be drawn from the WFS1 protein interaction network analyzed by STRING. The C-terminal region of wolframin is located on the cytoplasmic side of the ER membrane, adopting a folded confirmation. It can interact with the C-terminal region of the ER-localized Na + /K + ATPase beta1 subunit, which is important for subunit maturation. Na + /K + ATPase deficiency is known to be responsible for apoptosis and neural degenerative disease. If a similar association exists within the inner ear, amino acid substitutions may result in hearing loss in this way. The state of accumulation of misfolded and unfolded proteins in the organelle is ER stress. The unfolded protein response (UPR), also called the ER stress signaling network, can deal with ER stress in cells. Wolframin can negatively regulate the ER stress signaling network through interaction with the master regulators of the UPR (such as ATF6). Under normal conditions, WFS1 recruited ATF6α to an E3 ligase, HRD1, and the proteasome, prevents ATF6 activation and promotes ATF6 ubiquitination and proteasomal degradation. WFS1 also can reduce the expression of ATF6α target genes, for example HPSA5/GRP78/BiP and XBP-1. In patients with Wolfram syndrome, because of the variation of WFS1, ATF6 is hyperactivated, leading to dysregulated ATF6 signaling pathway. Wolframin can modulate the filling state of the ER Ca^2+^ store to participate in the regulation of cellular Ca^2+^ homeostasis. Once a variant occurs in WFS1, ER stress is strongly induced, and endolymphatic ion composition and homeostasis are disrupted, which leads to deafness. The C-terminal segment of wolframin protein in ER lumen bind to vesicular cargo proteins including proinsulin directly. The pathogenic variants in the domain may disrupt the interaction and result in abnormal accumulation of proinsulin in endoplasmic reticulum, which impede insulin secretion and proinsulin processing.

In conclusion, the bioinformatics analysis is useful to efficiently identify high-risk nsSNPs. Pathogenicity of some high-risk WFS1 nsSNPs has been confirmed by pedigree and genetic analysis, but further vivo and vitro functional studies are required to verify the accuracy of our methods.

## Supplementary Information

Supplementary Information.

## Data Availability

The data supporting the results reported in the article can be found in the Supplementary Information files. Web resources: dbSNP database, http://www.ncbi.nlm.nih.gov/projects/SNP/; ClinVar database, https://www.ncbi.nlm.nih.gov/clinvar; Deafness variation database, https://deafnessvariationdatabase.org/; SIFT, http://sift.jcvi.org/; PolyPhen-2, http://genetics.bwh.harvard.edu/pph2/; FATHMM, http://fathmm.biocompute.org.uk/inherited.html; Mutation Taster, http://www.mutationtaster.org/ChrPos.html; Mutation Assessor, http://mutationassessor.org/r3/; PROVEAN, http://provean.jcvi.org; VEST3, http://karchinlab.org/apps/appVest.html; MUpro, http://mupro.proteomics.ics.uci.edu; I-Mutant2.0, https://folding.biofold.org/i-mutant/i-mutant2.0.html; INPS-MD, http://inpsmd.biocomp.unibo.it; iStable, http://predictor.nchu.edu.tw/istable/indexSeq.php; ConSurf server, http://consurf.tau.ac.il; SOPMA, https://npsa-prabi.ibcp.fr/cgi-bin/npsa_automat.pl?page=npsa_sopma.html; TMHMM Server 2.0, https://services.healthtech.dtu.dk/service.php?TMHMM-2.0; Robetta, http://robetta.bakerlab.org/; HOPE, http://www.cmbi.umcn.nl/hope; Accessible Surface Area and Accessibility Calculation for Protein (ver. 1.2) online server, http://cib.cf.ocha.ac.jp/bitool/ASA/; STRING, https://cn.string-db.org/.
