# Supplementary material for: Mutation analysis of pathogenic non-synonymous single nucleotide polymorphisms (nsSNPs) in WFS1 gene through computational approaches
Source: Sci Rep. 2023 Apr 25;13:6774. doi: 10.1038/s41598-023-33764-1 (PMC10130013; doi:10.1038/s41598-023-33764-1)
Supplement: Supplementary file 1 — Supplementary Information. [file 41598_2023_33764_MOESM1_ESM.docx]

| **Supplementary Table S1 The Categorical prediction of high risk** | |
| --- | --- |
| **Name** | **Categorical prediction of high risk** |
| SIFT | D: Deleterious (≤0.05) |
| Polyphen2_HVAR | D: Probably damaging (≥0.909); P: Possibly damaging (0.447≤score≤0.909) |
| Polyphen2_HDIV | D: Probably damaging (≥0.957); P: Possibly damaging (0.453≤score≤0.956) |
| LRT | D: Deleterious |
| MutationTaster2 | A: Disease_causing_automatic; D: Disease_causing |
| MutationAssessor | H: High; M: Medium (H/M means functional) |
| FATHMM | D: Deleterious |
| PROVEAN | D: Deleterious |
| MetaSVM | D: Deleterious |
| MetaLR | D: Deleterious |
| VEST3 | score > 0.9 |
| REVEL | score > 0.9 |
| CADD | score > 25 |
| DANN | rankscore > 0.9 |
| FATHMM -MKL | rankscore > 0.9 |
| M-CAP | D: Deleterious |

| **Supplementary Table S2 The Clinical Significance of 13 high-risk nsSNPs** | | | | | |
| --- | --- | --- | --- | --- | --- |
| **Number** | **Substituent** | **SNP ID** | **ClinVar Accession** | **Disease Names** | **Clinical Significance** |
| 1 | G107R |  |  |  | Pathogenic* |
| 2 | G494R | rs760692398 | RCV001919107.1 | not provided | Uncertain-Significance |
| 3 | A684T | rs1412819148 | RCV001958686.1 | not provided | Pathogenic |
| 4 | **G695S** | rs1252460131 |  |  | **Not Reported in ClinVar** |
| 5 | G702S | rs71532862 | RCV001900340.3 | not provided | Pathogenic |
| 6 | L723P |  |  |  | Pathogenic* |
| 7 | P724L | rs28937890 | RCV000004767.3 | Wolfram syndrome 1 | Pathogenic |
|  |  |  | RCV000756934.10 | not provided | Conflicting-Interpretations-Of-Pathogenicity |
| 8 | R732H | rs149013740 | RCV000197209.20 | not provided | Conflicting-Interpretations-Of-Pathogenicity |
| 9 | G736S | rs71532864 | RCV001797328.4 | not provided | Pathogenic-Likely-Pathogenic |
| 10 | G736R |  |  |  |  |
| 11 | **E776K** | rs1421068689 |  |  | **Not Reported in ClinVar** |
| 12 | L829P | rs104893883 | RCV000004779.7 | Autosomal dominant nonsyndromic hearing loss 6 | Pathogenic |
|  |  |  | RCV000726781.6 | not provided | Likely-Pathogenic |
|  |  |  | RCV001267554.1 | Inborn genetic diseases | Likely-Pathogenic |
| 13 | P885L | rs372855769 | RCV001857186.2 | not provided | Pathogenic |
|  |  |  | RCV001849390.2 | Wolfram-like syndrome | Likely-Pathogenic |
|  |  |  | RCV000503566.5 | Wolfram syndrome | Likely-Pathogenic |

*The data of G107R and L723P was from the Deafness Variation Database.

| **Supplementary Table S3 WFS1 direct interacting proteins predictied by STRING** | | |
| --- | --- | --- |
| **Interacting protein** | **Protein details** | **Combined score** |
| XBP1 | X-box-binding protein 1; Functions as a transcription factor during endoplasmic reticulum (ER) stress by regulating the unfolded protein response (UPR). | 0.957 |
| ATF6 | Cyclic AMP-dependent transcription factor ATF-6 alpha; Transmembrane glycoprotein of the endoplasmic reticulum that functions as a transcription activator and initiates the unfolded protein response (UPR) during endoplasmic reticulum stress. Cleaved upon ER stress, the N-terminal processed cyclic AMP-dependent transcription factor ATF-6 alpha translocates to the nucleus where it activates transcription of genes involved in the UPR. | 0.924 |
| CISD2 | CDGSH iron-sulfur domain-containing protein 2; Regulator of autophagy that contributes to antagonize BECN1-mediated cellular autophagy at the endoplasmic reticulum. Participates in the interaction of BCL2 with BECN1 and is required for BCL2-mediated depression of endoplasmic reticulum Ca(2+) stores during autophagy. | 0.902 |
| ADCY8 | Adenylate cyclase type 8; This is a membrane-bound, calcium-stimulable adenylyl cyclase. May be involved in learning, in memory and in drug dependence (By similarity). | 0.838 |
| ATF6B | Cyclic AMP-dependent transcription factor ATF-6 beta; Transcriptional factor that acts in the unfolded protein response (UPR) pathway by activating UPR target genes induced during ER stress. | 0.837 |
| TMEM38A | Trimeric intracellular cation channel type A; Monovalent cation channel required for maintenance of rapid intracellular calcium release. May act as a potassium counter-ion channel that functions in synchronization with calcium release from intracellular stores. | 0.816 |
| ATP1B1 | Sodium/potassium-transporting ATPase subunit beta-1; This is the non-catalytic component of the active enzyme, which catalyzes the hydrolysis of ATP coupled with the exchange of Na(+) and K(+) ions across the plasma membrane. The beta subunit regulates, through assembly of alpha/beta heterodimers, the number of sodium pumps transported to the plasma membrane. | 0.799 |
| CDKAL1 | Threonylcarbamoyladenosine tRNA methylthiotransferase; Catalyzes the methylthiolation of N6- threonylcarbamoyladenosine (t(6)A), leading to the formation of 2- methylthio-N6-threonylcarbamoyladenosine (ms(2)t(6)A) at position 37 in tRNAs that read codons beginning with adenine | 0.762 |
| HHEK | Hematopoietically-expressed homeobox protein HHEX; Recognizes the DNA sequence 5'-ATTAA-3'. Transcriptional repressor. May play a role in hematopoietic differentiation. Establishes anterior identity at two levels; acts early to enhance canonical WNT-signaling by repressing expression of TLE4, and acts later to inhibit NODAL-signaling by directly targeting NODAL (By similarity). | 0.741 |
| THADA | Thyroid adenoma-associated protein; THADA, armadillo repeat containing; Belongs to the THADA family. | 0.732 |

Supplementary Figure S1: Combined figure. A. Schematic diagram of the exons of the WFS1 gene. B. Hypothetical structure of wolframin protein.


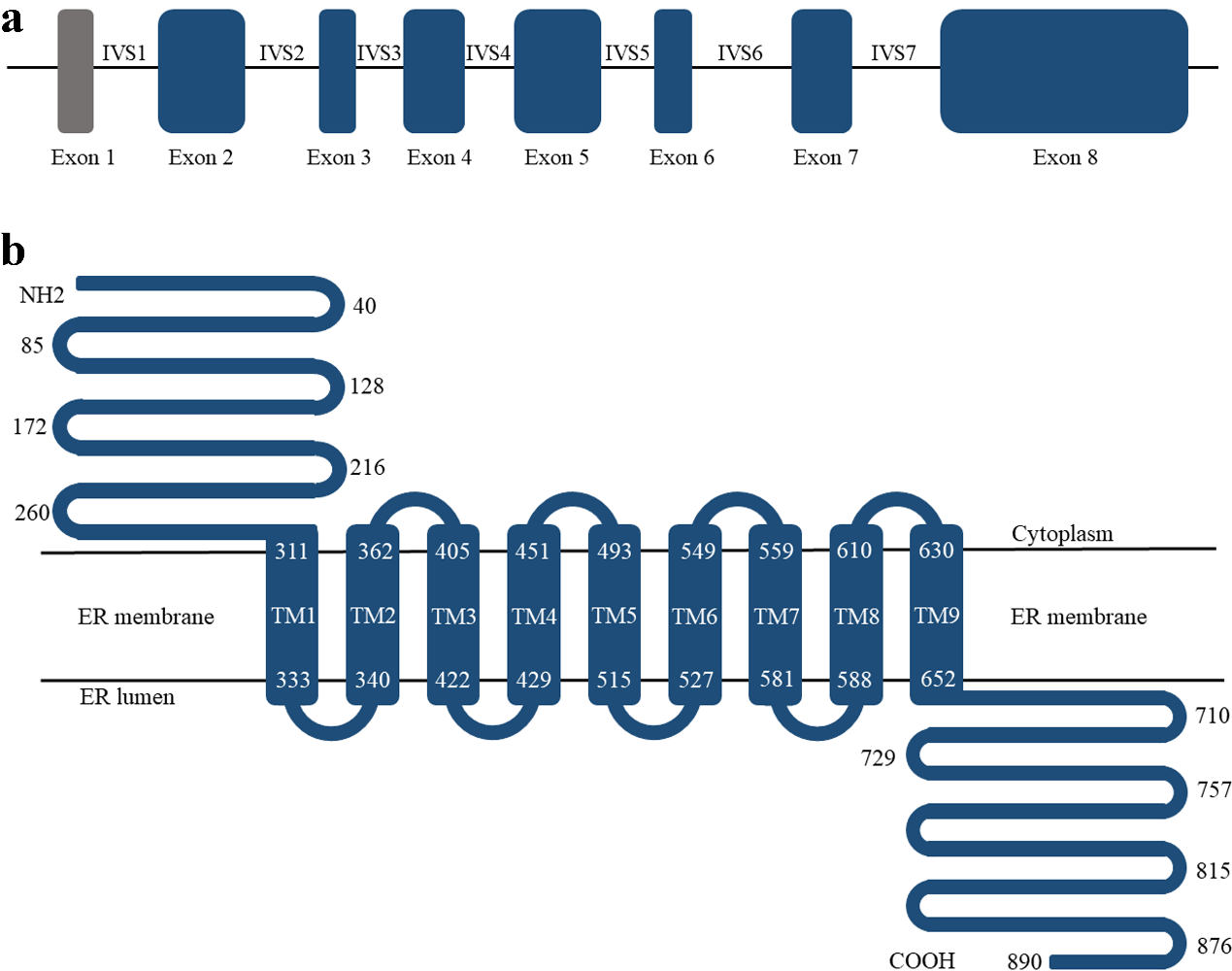


Supplementary Figure S2: The secondary structure of the wolframin protein predicted by SOPMA
